# Perianal tuberculosis presenting as a Fournier's gangrene

**DOI:** 10.1002/ccr3.8882

**Published:** 2024-05-02

**Authors:** Raquel Lalanda, Andreia Barão, Beatriz Draiblate, Ester Malcato, Hélder Matos, José Girão, Rosário Rosa, José Paulo Freire, Luís Miranda

**Affiliations:** ^1^ General Surgery Department—Centro Hospitalar Universitário Lisboa Norte EPE Hospital de Santa Maria Lisbon Portugal

**Keywords:** extensive necrotizing fasciitis, extrapulmonary tuberculosis, Fournier's gangrene, *Mycobacterium tuberculosis complex*, perianal tuberculosis, *Tuberculosis cutis orificialis*

## Abstract

**Key Clinical Message:**

In the setting of Fournier's gangrene, atypical clinical manifestations and complications in an immunocompetent patient warrant consideration of perineal tuberculosis as a potential underlying cause.

**Abstract:**

*Tuberculosis cutis orificialis* is a rare form of extrapulmonary tuberculosis that affects the perianal region. Fournier's gangrene is an aggressive necrotizing fasciitis that primarily involves the perianal area and external genitalia. A previously healthy 38‐year‐old man presented with a left perianal abscess. His condition deteriorated, leading to septic shock and multiorgan dysfunction syndrome. A CT scan displayed extensive necrotizing fasciitis. Treatment included broad‐spectrum antibiotics, numerous surgical perineal debridements, a transverse loop colostomy, and hyperbaric oxygen therapy. We believe the patient had pre‐existing asymptomatic, non‐diagnosed perianal tuberculosis, and a subsequent bacterial superinfection resulted in a perineal local abscess that progressed to severe Fournier's gangrene. The diagnosis of tuberculosis was confirmed through positive cultures and molecular identification in perineal biopsies. The patient experienced a complex clinical course with complications such as myocardial necrosis, acute respiratory distress syndrome, rhabdomyolysis with severe critical illness polyneuromyopathy and internal jugular thrombosis. Fournier's gangrene resulted in air dissection throughout the perineal fasciae, extending to the abdominal wall muscles resulting in an infected extraperitoneal spontaneous hematoma, probably caused by therapeutic anticoagulation. An extraperitoneal surgical drainage was performed. This case emphasizes the complexities in diagnosing and managing both perianal tuberculosis and Fournier's gangrene.

## Graphical abstract

Surgical treatment of perianal tuberculosis presenting as a Fournier’s Gangrene: surgical perineal debridement (A, B); vacuum therapy of the perineum—sponge with sorbact (C, D); clean perineum after vacuum therapy (E).

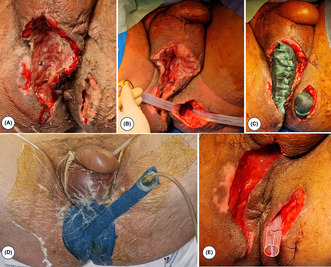

## 1 INTRODUCTION

Tuberculosis is an infectious granulomatous disease caused by *Mycobacterium tuberculosis* an alcohol‐acid‐resistant bacillus.^1^, ^2^

Pulmonary disease is the most frequent manifestation.^1^ Extrapulmonary tuberculosis accounts for at least 15% of all cases and gastrointestinal tuberculosis accounts for less than 1% of all cases.^1^, ^2^ The disease can involve any part of the gastrointestinal system.^1^

*Tuberculosis cutis orificialis*, involving anal and perianal areas,^1^, ^3^ is an extremely rare type of extrapulmonary tuberculosis comprising less than 1% of digestive tract incidence^2^ and in less than 0.0015% of all cases of tuberculosis.^4^ It is believed that its prevalence might be underestimated due to misdiagnosis as Crohn's or other granulomatous diseases.^2^ According to Tago et al. there were 58 cases of perianal tuberculosis described in the literature between 1970 and 2014.^2^

*Tuberculosis cutis orificialis* is caused by autoinoculation of the bacillus into the anal or perianal skin and mucous membranes in patients with active gastrointestinal tuberculosis,^1^, ^3^ hematogenous spread of a preexisting tuberculosis infection in another organ (e.g., pulmonary focus) or tissue,^4^, ^5^ ingestion of sputum containing active pulmonary disease bacilli^1^, ^4^ or lymphatic dissemination of intestinal or genitourinary disease from regional lymph nodes.^1^ After implantation into the mucosa, the microorganism usually causes an ulcer that is further infected with enteric bacteria.^5^

A Fournier's gangrene is a necrotizing fasciitis localized on the external genital organs and perianal region.^6^ It can reach 90% mortality rate, if not promptly diagnosed and treated with antibiotic therapy and an emergency surgical debridement.^6^, ^7^

The association of both conditions is rare, especially in immunocompetent patients.

## 2 CASE HISTORY

A 38‐year‐old male with no known medical conditions presented to a private hospital with severe perianal pain. He was diagnosed with a perianal abscess and prescribed doxycycline 100 mg twice a day. Three days later, he returned to our emergency department (ED) with worsening perianal pain and additional pain in the lower abdominal quadrants.

**FIGURE 1:**
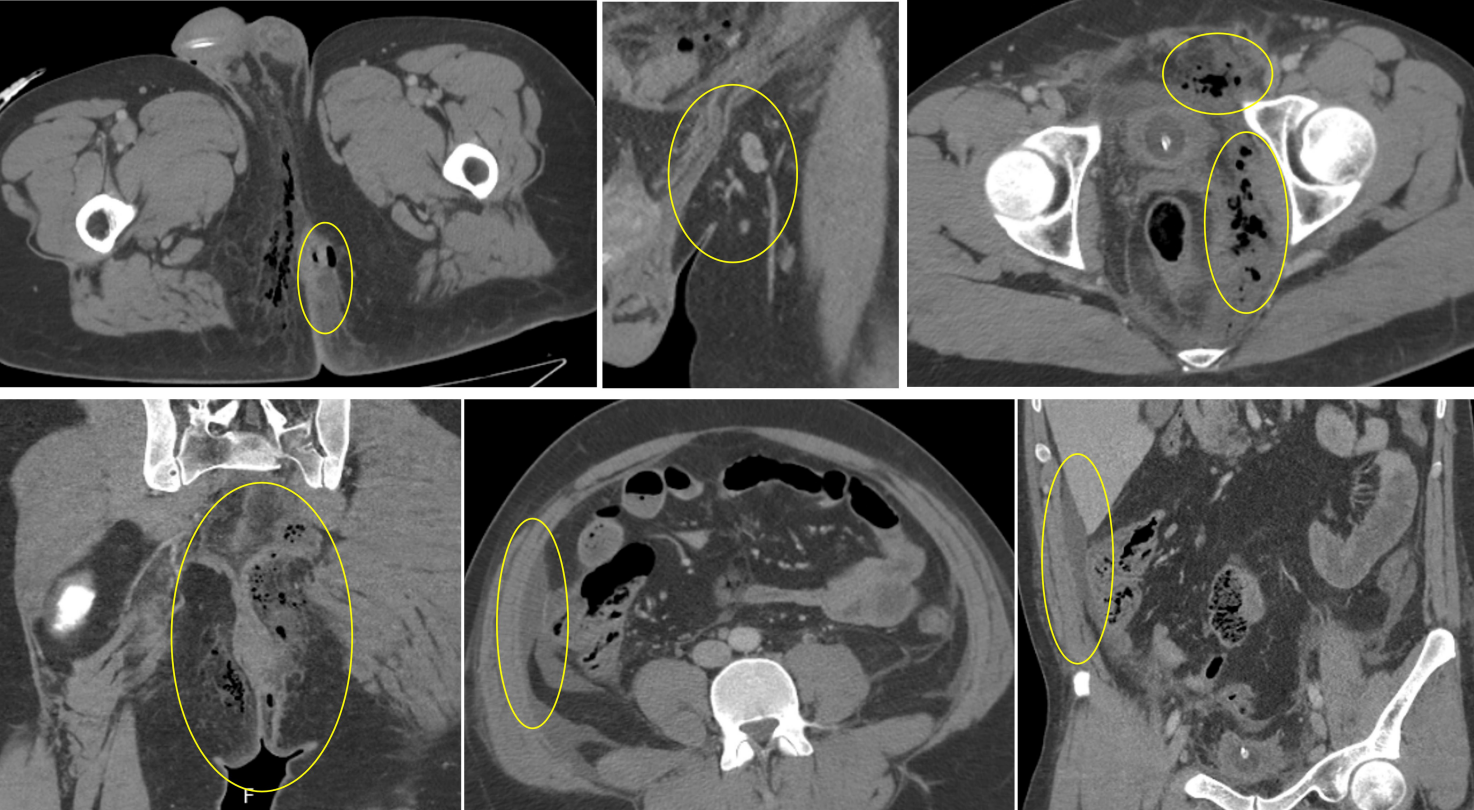
Abdominal CT scan shows a 6.3 × 2.6 × 4.9 cm^3^ left perianal abscess with gas accumulation in the space of Retzius, base of the penis, intergluteal region, and ischiorectal fossa. Additionally, there was free fluid in the pelvic region and along the parietocolic gutters, as well as enlarged lymph nodes in the left external iliac region measuring approximately 15 mm in short‐axis diameter. Findings consistent with extensive necrotizing fasciitis, specifically Fournier's gangrene.

**FIGURE 2:**
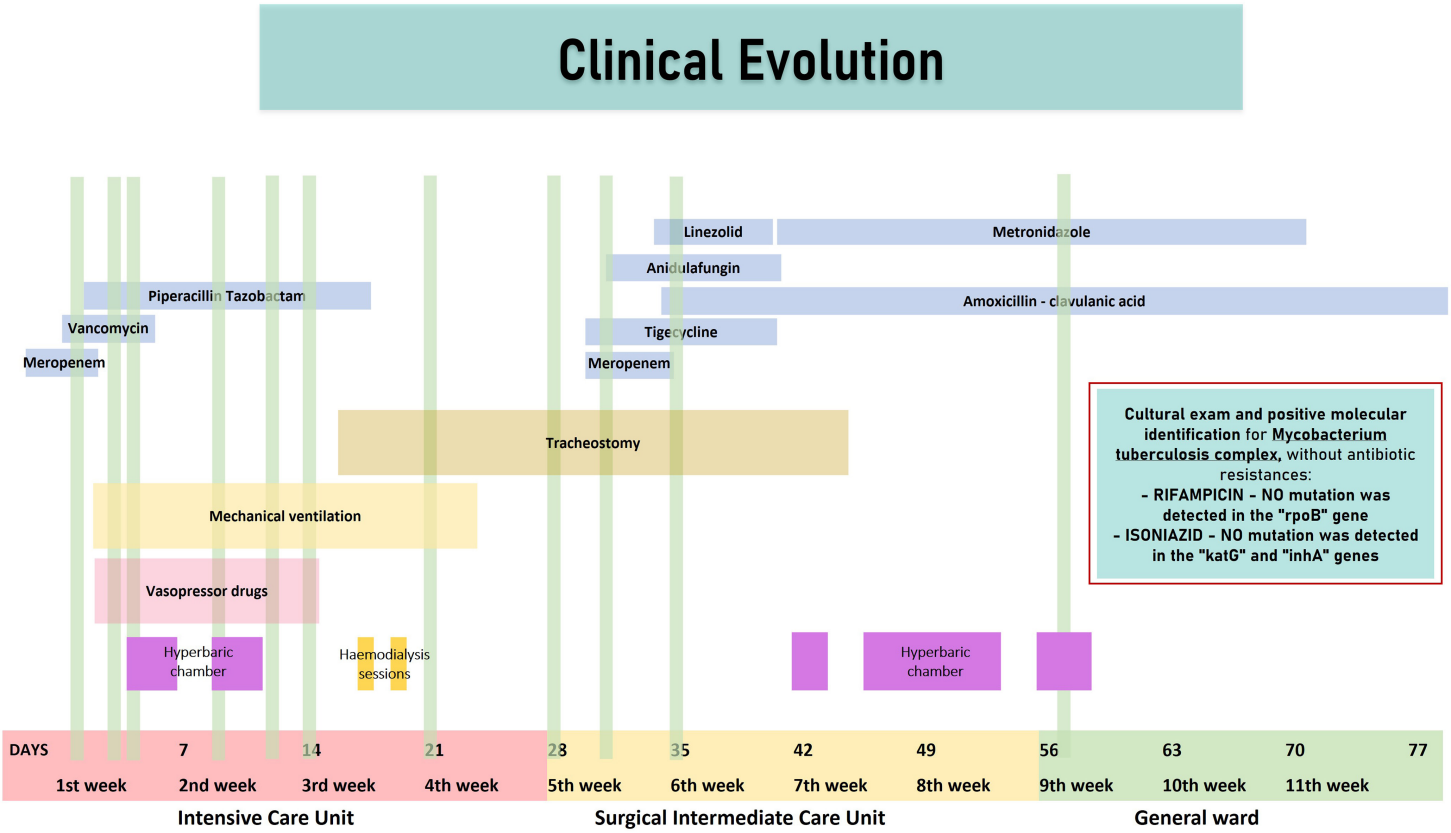
Representation of patient's clinical evolution; vertical green strips represent surgical debridement at the operatory room. The culture results, revealing mycobacterium tuberculosis complex, were obtained 1 month post‐sample harvest.

**FIGURE 3:**
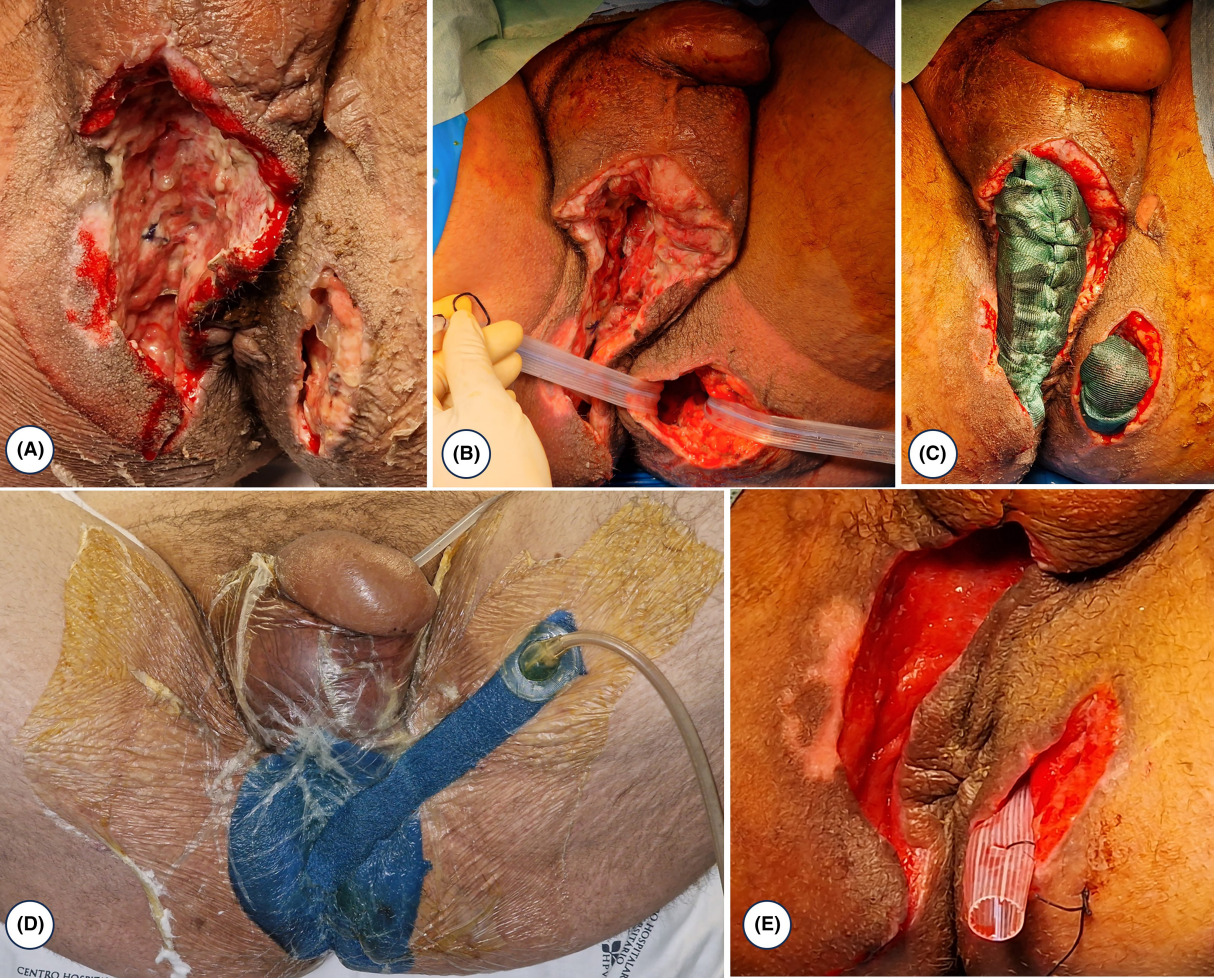
Surgical perineal debridement (A, B); vacuum therapy of the perineum—sponge with sorbact (C, D); clean perineum after vacuum therapy (E).

Upon physical examination, the patient presented with hypotension and slight tachycardia, along with fever, left perianal edema, mild tenderness, and perianal pain. Initial examination in the ED revealed a white blood cell count of 4.5 × 10^9^/L, C‐reactive protein (CRP) level of 59.7 mg/dL, procalcitonin (PCT) level of 9.9 μg/L, serum creatinine level of 2.89 mg/dL, and urea level of 128 mg/dL. An abdominal CT scan (Figure 1) showed a 6.3 × 2.6 × 4.9 cm^3^ left perianal abscess with gas accumulation in the space of Retzius, base of the penis, intergluteal region, and ischiorectal fossa. Additionally, there was free fluid in the pelvic region and along the parietocolic gutters, as well as enlarged lymph nodes in the left external iliac region measuring approximately 15 mm in short‐axis diameter. These findings were consistent with extensive necrotizing fasciitis, specifically Fournier's gangrene. The patient's condition worsened (Figure 2), leading to septic shock and multi‐organ dysfunction syndrome, urging transfer to the intensive care unit (ICU) of our hospital. He received broad‐spectrum antibiotics and underwent surgical perineal debridement (Figure 3), as well as the creation of a temporary transverse loop colostomy.

## 3 METHODS

The patient remained in the ICU for 1 month due to multi‐organ dysfunction syndrome resulting from septic shock. Upon admission, he experienced hemodynamic failure requiring vasopressor support. Extensive myocardial necrosis was observed, as evidenced by a peak troponin T level of 43,000 ng/mL, without other clinical, electrocardiographic or echocardiographic signs except for a single episode of atrial fibrillation. This condition improved during the first 2 weeks in the ICU. The patient also developed acute respiratory distress syndrome, which gradually resolved over the course of 4 weeks. Weaning from invasive mechanical ventilation was challenging due to severe critical illness polyneuromyopathy, necessitating a tracheostomy. Additionally, the patient experienced extensive rhabdomyolysis (creatine kinase level of 35,000 U/L) accompanied by non‐oliguric acute kidney injury, requiring two sessions of hemodialysis. He also developed extensive thrombosis in the right internal jugular vein, leading to the initiation of therapeutic anticoagulation.

During the patient's ICU stay, the septic focus was managed with antibiotic therapy and multiple surgical perineal debridements (performed seven times), along with perineum vacuum therapy. Additionally, six sessions of hyperbaric oxygen therapy were provided. As the inflammatory parameters decreased and the CT scan showed improvement, and because the microorganisms isolated from the perineal pus (*Bacteroides fragilis*, *Peptostreptococcus micros*, *Prevotella intermedia*, *Pseudomonas aeruginosa*, and *Escherichia coli*) did not exhibit significant antibiotic resistance, antibiotic therapy was discontinued in the third week.

**FIGURE 4:**
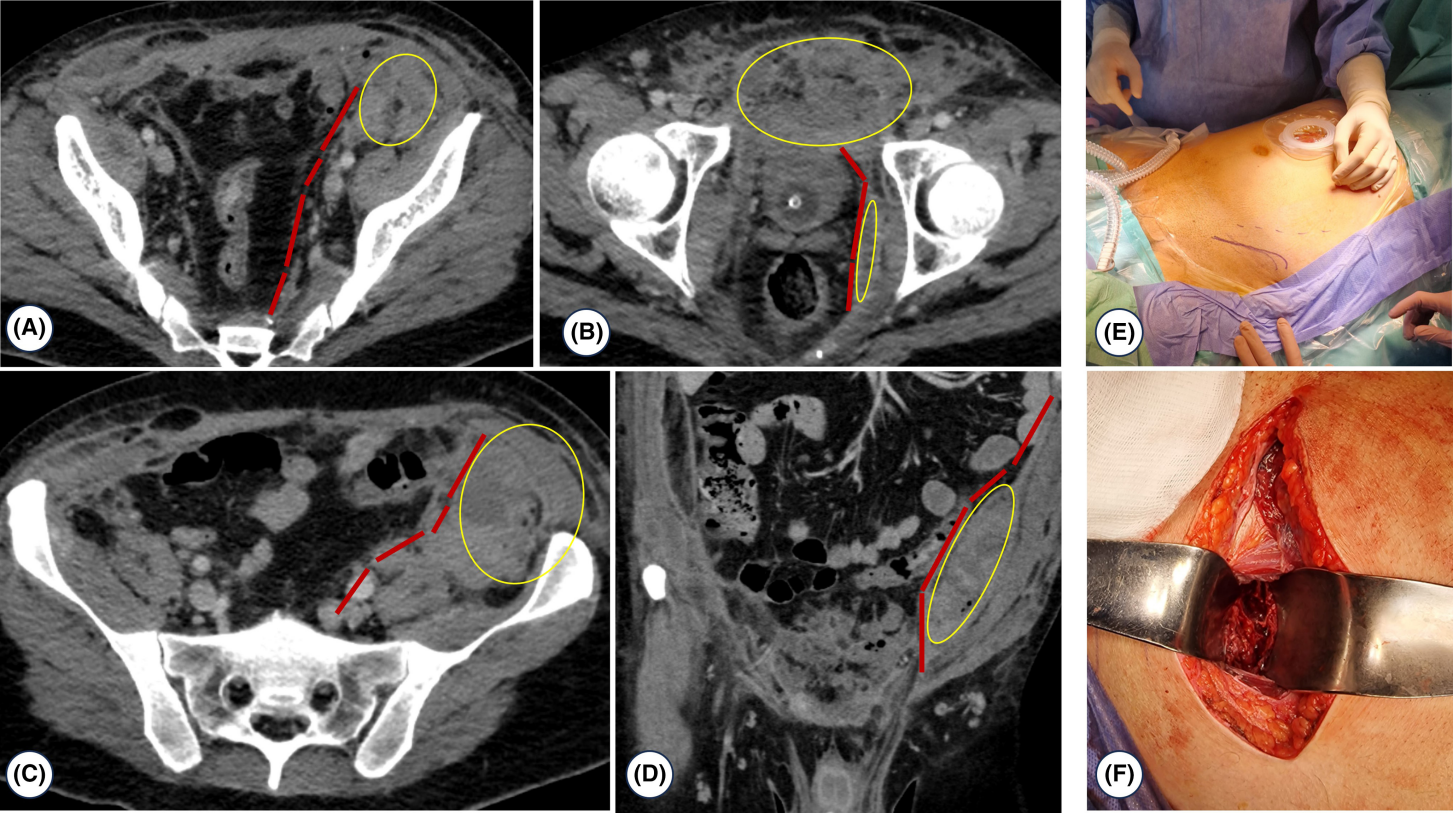
CT scan 4 weeks later (A, B, C, and D): Fluid‐air collections in the abdominal wall, space of Retzius, perirectal area with pelvic extension, and a new collection near the left iliac fossa. Fournier's gangrene resulted in air dissection throughout the perineal fasciae, extending to the fasciae of the abdominal wall muscles (necrotizing fasciitis spread through fascial planes—spread cranially through the space of Retzius). The infected extraperitoneal collection in the left iliac fossa was attributed to a spontaneous infected hematoma caused by therapeutic anticoagulation; an extraperitoneal surgical drainage of this infected organized hematoma was performed (E, F); red dashed lines correspond to the peritoneum; yellow circles correspond to the fluid‐air collections.

After 4 weeks, the patient was transferred to a surgical intermediate care unit. He no longer required vasopressor therapy, was breathing spontaneously through the tracheostomy without supplemental oxygen, and was slowly recovering from acute kidney injury. Despite local improvement of the perineum, inflammatory parameters increased progressively, and fever recurred. New cultures and perineal and ischioanal fossa biopsies were obtained, and broad‐spectrum antibiotic therapy was initiated. CT scan findings worsened, revealing fluid‐air collections in the abdominal wall, space of Retzius, perirectal area with pelvic extension, and a new collection near the left iliac fossa (Figure 4). We believe that Fournier's gangrene resulted in air dissection throughout the perineal fasciae, extending to the fasciae of the abdominal wall muscles. The infected extraperitoneal collection in the left iliac fossa was attributed to a spontaneous infected hematoma caused by therapeutic anticoagulation. An extraperitoneal surgical drainage of this infected organized hematoma was performed, resulting in excellent outcome and significant patient improvement.

## 4 CONCLUSION AND RESULTS

**FIGURE 5:**
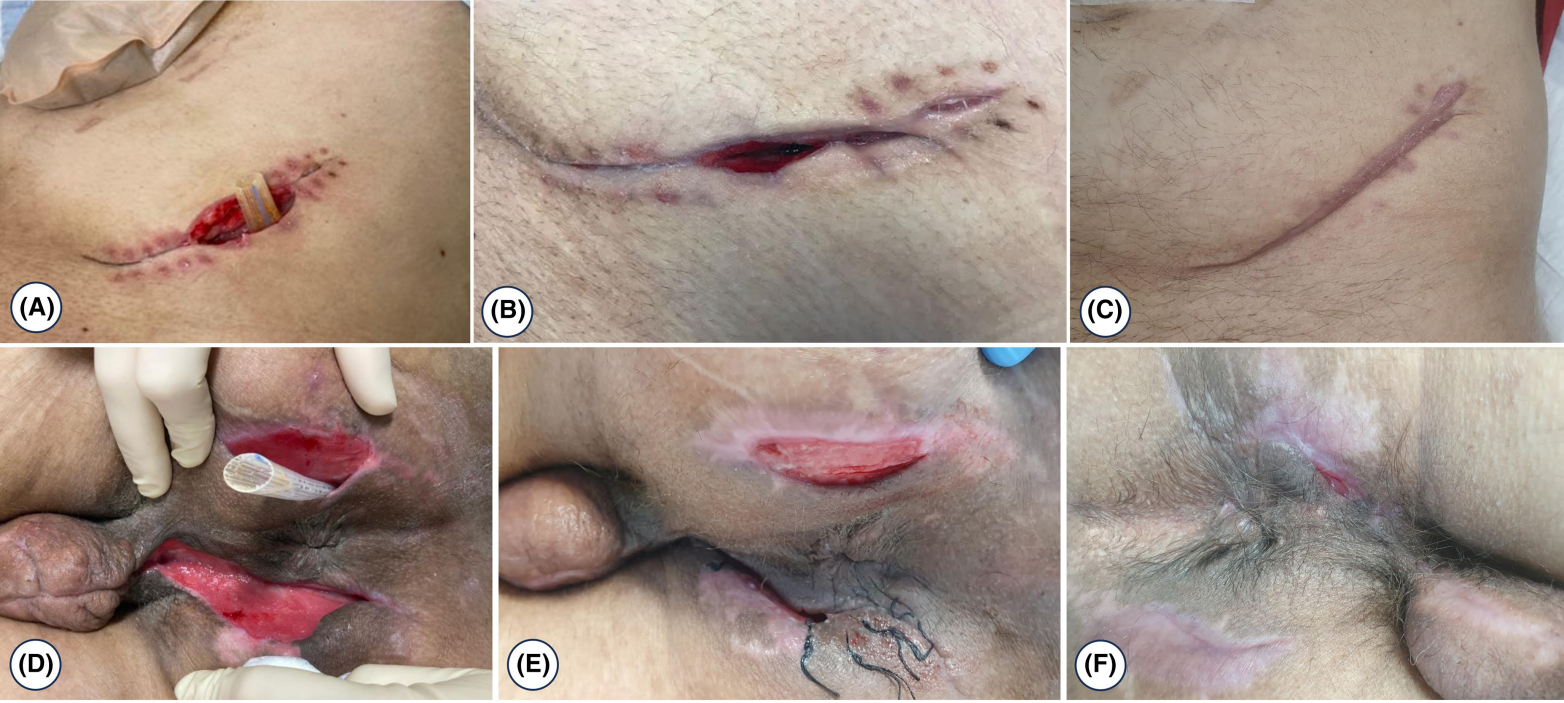
Progressive healing of the abdominal lesion (A, B, C) and perineal lesions (D, E, F) was observed, aided by the use of vacuum therapy with sorbact and hyaluronic acid.

The patient's clinical condition improved, and inflammatory parameters markedly decreased. Given the presence of only minimal residual collections on CT, it was decided to discontinue antibiotic therapy consisting of amoxicillin and clavulanic acid and metronidazole. The patient underwent an additional 10 sessions of hyperbaric oxygen therapy and was discharged to the general ward. Subsequently, the culture examination and molecular identification of perineal biopsies showed the presence of *Mycobacterium tuberculosis complex* without any detected resistance. The patient currently has complete healing of the perineal and abdominal lesions (contribution of the use of vacuum therapy with sorbact (Figure 3), and hyaluronic acid) (Figure 5). After consultation with the Infectious Disease specialty, the patient received 6 months of antituberculosis treatment.

## 5 DISCUSSION

Despite our patient being young and healthy, he experienced an unusual clinical course characterized by a prolonged hospital stay, an aggressive infection, multiple complications and setbacks, including initial septic shock, and severe critical illness polyneuromyopathy.

Fournier's gangrene is characterized by a rapid spread of the infection process in the depths of tissues along the fascia and the delay of external skin changes leading to an underestimation of the disease's severity.^6^

Fournier's gangrene usually appears in patients with diabetes, obesity, malignant neoplasms, or immunocompromised patients.^6^ Our patient, who had no previous known medical conditions, received appropriate antibiotic therapy for all isolated microorganisms, with the exception of the first empiric treatment administered before admission to our hospital.

The most common computed tomography findings of Fournier's gangrene are fascial gas with or without surrounding soft‐tissue inflammatory change, muscle or fascial edema, and subcutaneous edema.^7^ Computed tomography can be useful for determining the extent of involved fascial planes and preoperative planning.^7^ Most operations for Fournier's gangrene do not require a laparotomy which is why identifying extraperitoneal spread along the space of Retzius and intraperitoneal extension is particularly important.^7^ In this case understanding the spread along the fascial planes allowed us to drain the infected extraperitoneal collection of the left iliac fossa using an extraperitoneal approach.

Patient was extensively investigated. There was no previous history of active tuberculosis, other diseases, or known immunodeficiencies and he was not taking any immunosuppressive therapy. He tested negative for P24 Antigen, AntiHIV 1 and 2 antibodies, HBs Antigen, Anti‐HCV Antibody, and anti‐*Treponema pallidum* antibody. Both his immunoglobulin assay and protein electrophoresis were normal. Ziehl‐Neelsen stains of both sputum and perineal pus were negative, with no imaging findings indicative of pulmonary tuberculosis. There was no history of travel to countries with a high incidence of tuberculosis, or of rectal foreign body insertion as part of sexual practices.

The diagnosis was only established after a mycobacterial culture of perineal biopsies came back positive for *Mycobacterium tuberculosis complex*. The results were further confirmed with a molecular identification. Nowadays PCR‐based assay for *Mycobacterium tuberculosis* has the highest sensitivity (79.4%).^1^ Conventional culture is the gold standard for detection of mycobacterium tuberculosis, but it is time‐consuming, taking at least 3–4 weeks to have results.^1^, ^8^, ^9^ Direct test with the detection of acid‐fast bacilli by microscopy (Ziehl–Neelsen staining method) is negative in 75% of patients with extra pulmonary tubercular disease.^9^, ^10^

We believe the patient had pre‐existing asymptomatic, non‐diagnosed perianal tuberculosis, and a subsequent bacterial superinfection resulted in a perineal local abscess that progressed to severe Fournier's gangrene.

After establishing the diagnosis of perianal tuberculosis, a thorough search for the origin and involvement of other organs and tissues should be undertaken.^1^ According to Tago et al., only 62.7% cases were sufficiently investigated and included data on intestinal examination.^2^ In the reported case, CT scans of the thorax, abdomen, pelvis, and spine were not able to identify any other focus of tuberculosis. The most frequently involved site of the intestinal tract is the ileocecal region, accounting for more than 85% of the gastrointestinal tract tuberculosis.^1^, ^8^ Patient performed a colonoscopy which also ruled out intestinal involvement of the disease.

Perianal tuberculosis usually manifests in the fourth decade of life and is more commonly found in men,^1^ such as in our case. One literature review showed that only 18.6% of the patients had underlying diseases related to immunodeficiency.^2^ We also report a case of an immuno‐competent patient.

It may arise without any previous or active lung infection,^2^ such as in this case. Perianal fistulas with a prolonged and relapsing course despite proper surgical management are the most frequent symptom of perianal tuberculosis (80%–91% of cases).^1^, ^9^

The first‐line treatment is conventional anti‐mycobacterium therapy for at least 6 months after surgery.^11^, ^12^ For complicated disease presentations, such as this case, an extension of the antimycobacterium treatment course to 9–18 months is mandatory.^12^

Antimicrobial susceptibility testing should be performed to select the best treatment to eradicate tuberculosis and prevent the emergence of resistant cases.^1^

As far as we know, this is the first reported case of using hyperbaric oxygen therapy to help treat Fournier's gangrene associated with perianal tuberculosis. Studies reveal that hyperbaric oxygen therapy can be used as an additional treatment in Fournier's gangrene.^13^ Further studies or even RCTs are challenging to carry out due to the rareness and complexity of the disease and restricted availability of this treatment.^13^

Patients should be followed‐up until the perianal tuberculosis fistulas have completely healed.^14^ Our patient had 6 months follow‐up. He was then oriented to surgery to close the stoma.

In summary, tuberculosis should be considered as a potential causative agent in cases of non‐healing or recurrent perianal fistula.^2^ We present a case of Fournier's gangrene in an immunocompetent patient with an unusual clinical course, marked by a prolonged hospital stay, an aggressive infection, multiple complications and setbacks. Due to the absence of an identifiable primary focus of tuberculosis, the diagnosis of a perianal tuberculosis was a challenge.

## AUTHOR CONTRIBUTIONS

**Raquel Lalanda:** Conceptualization; investigation; methodology; resources; software; visualization; writing – original draft; writing – review and editing. **Andreia Barão:** Validation; visualization. **Beatriz Draiblate:** Validation; visualization. **Ester Malcato:** Validation; visualization. **Hélder Matos:** Validation; visualization. **José Girão:** Validation; visualization. **Rosário Rosa:** Conceptualization; formal analysis; methodology; project administration; supervision; validation; visualization; writing – review and editing. **José Paulo Freire:** Validation; visualization. **Luís Miranda:** Validation; visualization.

## FUNDING INFORMATION

This research received no specific grant from any funding agency in the public, commercial, or not‐for‐profit sectors.

## CONFLICT OF INTEREST STATEMENT

All authors declare no conflict of interests.

## ETHICS STATEMENT

The authors affirm the accuracy of the report's content. We are committed to patient welfare, transparency, and accountability throughout this publication.

## CONSENT

Written informed consent was obtained from the patient to publish this report in accordance with the journal's patient consent policy.

## Data Availability

The data that support the findings of this study are available from the corresponding author upon reasonable request.
